# DNA repair and damage pathways in mesothelioma development and therapy

**DOI:** 10.1186/s12935-022-02597-9

**Published:** 2022-05-02

**Authors:** Faezeh Malakoti, Niloufar Targhazeh, Erfan Abadifard, Reza Zarezadeh, Sahar Samemaleki, Zatollah Asemi, Simin Younesi, Reza Mohammadnejad, Seyed Hadi Hossini, Ansar Karimian, Forough Alemi, Bahman Yousefi

**Affiliations:** 1 Department of Biochemistry and Clinical Laboratories, Faculty of Medicine, Tabriz University of Medical Sciences, Tabriz, Iran; 2 School of Medicine, Tehran University of Medical Sciences, Tehran, Iran; 3 Students’ Scientific Research Center (SSRC), Tehran University of Medical Sciences, Tehran, Iran; 4 Department of Immunology, Faculty of Medicine, Hamadan University of Medical Sciences, Hamadan, Iran; 5 Research Center for Biochemistry and Nutrition in Metabolic Diseases, Kashan University of Medical Sciences, Kashan, Iran; 6 Schoole of Health and Biomedical Sciences, RMIT University, Melbourne, Vic Australia; 7 Cellular and Molecular Biology Research Center, Health Research Institute, Babol University of Medical Sciences, Babol, Iran

**Keywords:** Malignant mesothelioma, Malignant peritoneal mesothelioma, DNA damage repair, Signaling pathways, BRCA1 associated protein 1, BAP1

## Abstract

Malignant mesothelioma (MMe) is an aggressive neoplasm that occurs through the transformation of mesothelial cells. Asbestos exposure is the main risk factor for MMe carcinogenesis. Other important etiologies for MMe development include DNA damage, over-activation of survival signaling pathways, and failure of DNA damage response (DDR). In this review article, first, we will describe the most important signaling pathways that contribute to MMe development and their interaction with DDR. Then, the contribution of DDR failure in MMe progression will be discussed. Finally, we will review the latest MMe therapeutic strategies that target the DDR pathway.

## Introduction

Malignant mesothelioma (MMe) is an aggressive high lethal cancer that mostly arises from mesothelial cells in the pleural and peritoneum [1]. Although malignant peritoneal mesothelioma (MPeM) is uncommon [2], malignant pleural mesothelioma (MPM) is also a rare human cancer and it is the most common type of MMe by 80% [3]. Exposure to chemical components is a big health issue around the world [4] and in this regard, it has been widely investigated that asbestos exposure is the main cause of MMe progression. Genomic damages (mostly due to this exposure), the activation of cell signalings, and defects in the DNA damage repair (DDR) system are the most important factors leading to MMe. On this basis, CDKN2A/ARF, neurofibromin 2 (NF2), and BRCA1 associated protein 1 (BAP1) are frequently mutated genes in MPMs [5]. All these three genes are introduced as tumor suppressor genes whose mutations are related to cancer development. Furthermore, the dysregulation of survival pathways has been investigated and the theory of their significant contribution to MMe development was also proved [6, 7]. Besides, it has been the subject of encouraging several types of research that there is a link between various repair machinery errors and MMe development. Surgery, chemotherapy, and radiotherapy are considered the selective ways of treatment in these patients. However, DDR has become the center of attention in recent years and is most likely to open new avenues in offering therapies for patients with MMe. So, targeting this system like implementing PARP inhibitors can decrease cancer cell repair and survival ability [8]. Taken together, a combination of therapies has been suggested. In this review, we will bring the current understanding of some important signaling pathways involved in MMe progression. Then we discuss the role of DDR in MMe development and finally outline new strategies to reduce MMe mortality through DDR targeting therapy.

### Mesothelioma biology and involved signaling pathways

The dysregulation of major signaling pathways has been the subject of a huge number of studies investigating their possible role in cancers development and tumorigenesis. On this basis, multiple studies have reported higher expression levels and activity of Hedgehog (HH), mTOR, MAPK, and Calcium signaling pathways that are associated with the worst outcome and survival in MMe patients (Table 1). In the following, we will further discuss the role of these survival pathways in MMe.

**Table 1:** The effects of signaling path-ways inhibitors on MMe

Signaling	Inhibitor	Effect	Refs.
HH signaling	HhAntag (SMO antagonist)	35% ↓tumor volume and 32% ↓YAP protein and ↓SOX2 and survivin, ↓tumor growth	[14]
	Vismodegib	Gli1↓, Hhip↓, Ptch1↓, ↓tumor growth and ↓proliferation	[17]
	GDC-0449	↓proliferation	[19]
	GANT61	↓proliferation	[19]
	Cyclopamine	GLI1↓, PTCH1↓, SHH↓, SMO↓, and ↓proliferation	[20]
PI3K/mTOR signaling	BEZ235	↓cell viability and ↓proliferation	[27]
	Apitolisib with Platinum-pemetrexed chemotherapy	↓tumor volume, ↓symptoms, ↓CA-125	[28]
	BEZ235 and BYL719 with palbociclib	↓proliferation and↑ cellular senescence	[32]
	NVP-BEZ235 and GDC-0980 with Chloroquine	↓autophagy and ↓ cell death resistance	[33]
MAPK/ERK signaling and AKT/mTOR signaling	Pirfenidone alone or with cisplatin	↓proliferation	[42]

### Hedgehog and YAP signaling pathways

**Fig. 1:**
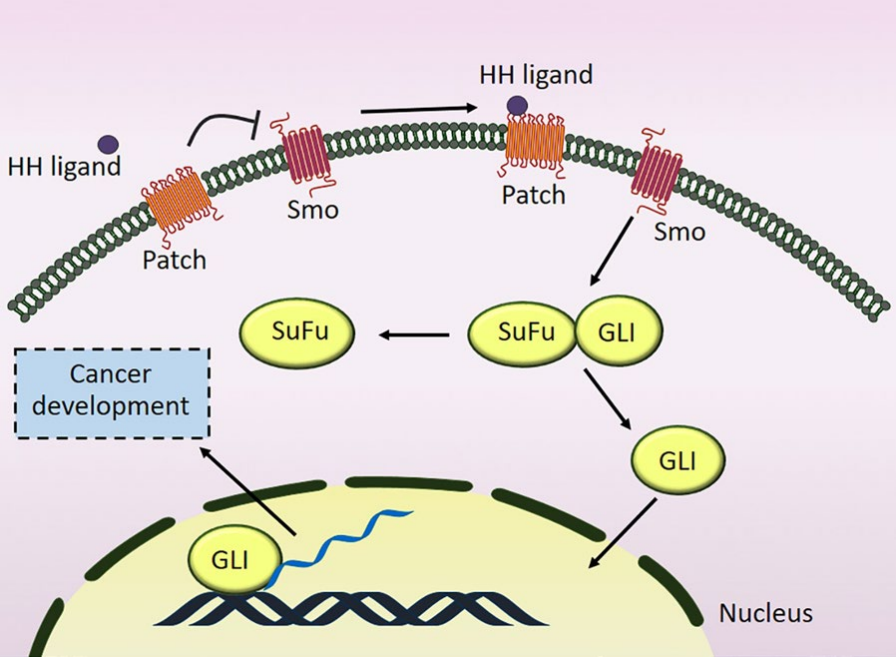
A schematic diagram depicting the effect of Hedgehog signaling on the cancer development. Following the binding of HH ligands to the PTCH receptor, the inhibitory effect of PTCH on SMO protein is removed, leading to GLI-SuFu separation and GLI translocation to the nucleus and finally protein expression. *HH* Hedgehog, *SMO* Smoothened, *SuFu* suppressor of fused protein

Hedgehog (HH) signaling is introduced as an embryonic/developmental signal transduction pathway that appears to play essential roles in embryonic development through the modulation of proliferation, differentiation, and body patterning as well as tissue homeostasis upon injuries in differentiated cells. Furthermore, this signaling pathway has been the subject of many studies investigating its possible influential function in the progression and maintenance of multiple types of cancers [9]. There are some HH signaling core components, including PTCH receptors, HH ligands, Smoothened (SMO), suppressor of fused protein (SUFU), and GLI, which are vital for signal initiation, transduction, and transcriptional regulation [10]. GLI as a HH signaling effector binds to GLI responsive genes to modulate their transcription to control cell fate and proliferation, But SUFU as the main GLI repressor maintains it in the cytoplasm. Moreover, transmembrane protein SMO is a G protein-coupled receptor and has a vital role in HH signal transduction from the membrane to the cytoplasm. When HH ligands are not present, the PTCH receptor interacts with SMO and suppresses its function. When HH ligands (Sonic HH (SHH), Desert HH (DHH), and Indian HH (IHH)) bind to the PTCH receptor, they can remove the inhibitory effect of PTCH on SMO protein. Now, activated-SMO initiates the transduction of the signal to the SUFU-GLI complex located in the cytoplasm. So, SMO leads to the separation of GLI from the complex and translocation from the cytoplasm to the nucleus. Finally, GLI as a transcription factor provokes the expression of many proteins involved in cancer development [11, 12] (Fig. 1). Although HH signaling has an imperative role in embryonic mesothelial development [13], it is inactive in most adult tissues, like the mesothelium. However, the HH pathway is one of the top ten pathways which dysregulated in MPM. Indeed, major HH components like GLI1, GLI2, and PTCH1 are highly expressed in MMe cell lines and tumors compared with normal mesothelial cells and adjacent tissues. Moreover, denovo sequencing on 7 human MMe cell lines detected 35 SNPs which are most frequently for GLI2, KIF7, and SMO. This data highlights the importance of the HH pathway in MMe [11].

Therefore, various studies have recently reported that HH target genes overexpression in MPM is associated with the worst clinical outcome. Higher gene expression of GLI1, SMO, YAP, and SHH are detected in MPM patients which are characterized by short overall survival [14, 15].

Moreover, HH signaling activates in both tumor and tumor microenvironments. Thus, not only autocrine HH signaling but also HH signaling in the stroma of the tumor microenvironment plays a significant role in tumor growth [16]. Solutions are required to overcome these barriers to improve MMe therapy. For example, in a study, although in vitro vismodegib treatment did not change HH genes expression and cell death, this therapy could prohibit HH signaling and tumor growth in MPM in vivo. On this basis, experimental data show that the downregulation of HH cascade by inhibitors reduces tumor volume [17].

Furthermore, it has been suggested that there is a cross-talk between HH signaling and YAP signaling in MMe. YAP is a transcriptional regulator located in the nucleus in its active form and provokes cancer stem cell attributes, proliferation, chemoresistance, and metastasis in a variety of cancers including MMe. In general, above 70% of MPM is characterized by a high protein level of YAP [18]. In vivo and in vitro investigations on nude mice uncovered that treatment with pharmacologic HhAntag (SMO antagonist) inhibits HH signaling, YAP, SOX2, and survivin. Pharmacologic HhAntag treatment resulted in about 35% tumor volume reduction after 2 weeks and a 32% decrease observed in the active nuclear form of YAP protein and also a significant decline in the protein level of stem cell marker SOX2 and survivin (a YAP down-stream effector). Overall two facts were obtained from this study; first, there is a positive correlation between HH signaling and maintaining YAP protein stability and function in SOX2 expression as well as tumor growth. Second, SMO inhibition is an effective strategy to reduce MMe tumor growth [14]. In this regard, Vismodegib, one of the SMO antagonists, appears to downregulate the expression level of Gli1, Hedgehog Interacting Protein (Hhip), and Ptch1 especially in the stroma to suppress HH signaling and tumor growth. Although this drug reduces proliferation, it doesn’t influence cell death [17].

Moreover, HH signaling inhibition decreases cell proliferation by about 40–60%. Generally, the most effective strategy for HH suppression is implementing pharmacologic SMO antagonists (GDC-0449) or Gli1/2 antagonists (GANT61). ATO and ITRA are two FDA-approved drugs that can inhibit HH signaling in MPM cells [19]. Moreover, an experimental in vitro study shows that cyclopamine as a HH signaling repressor can inhibit the gene expression of GLI1, PTCH1, SHH, and SMO and subsequently repress proliferation in MMe cell lines. In this study, combinational therapy with cisplatin and pemetrexed has been also suggested [20]. The uncertainty surrounding the effectiveness of MMe co-treatment with HH inhibitors and chemotherapeutic agents is one of the serious gaps in this field of research and future examinations are required.

As a result, the significant effect of HH signaling in MMe cell proliferation has been proved in multiple studies. Thus, this signaling pathway can be a potential option for targeting and combinational therapy.

### PI3K/AKT/mTOR signaling pathway

**Fig. 2:**
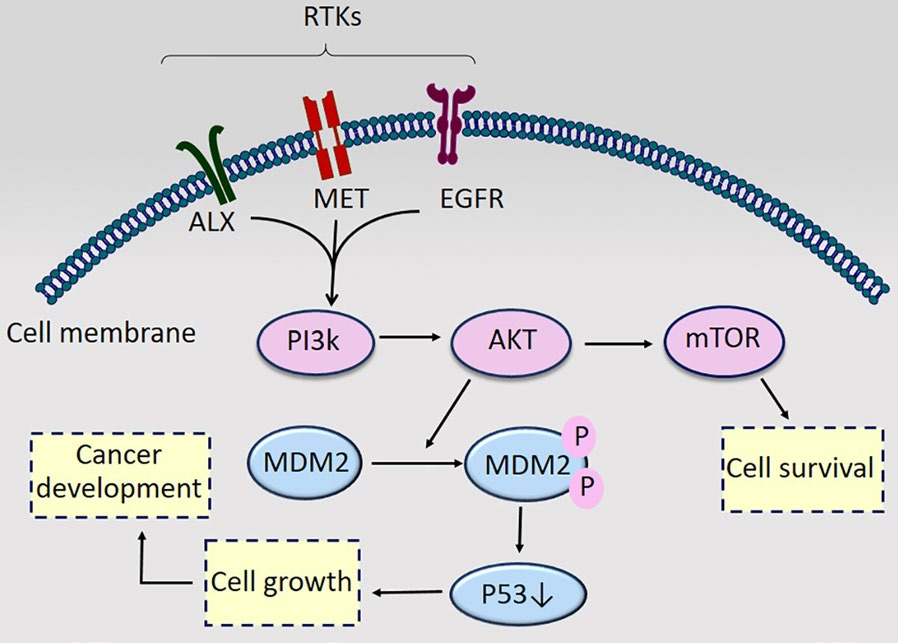
PI3K/AKT/mTOR signaling in mesothelioma progression. RTKs’ overexpression activates PI3K/AKT/mTOR signaling, leading to cell survival and MDM2 activation which mediates P53 degradation and cell growth. *RTKs* Receptor tyrosine kinases

mTOR is a serine/threonine kinase from the phosphoinositide-3-kinase-related kinase (PIKK) family. This protein kinase as a downstream component of the PI3K/AKT pathway has an imperative role in cell growth, proliferation, metabolism, migration, and overall tumorigenesis of multiple types of cancers [21]. Moreover, mTOR has a regulatory role in the modulation of proteins involved in DDR like p53, p21, and MDM2 (Fig. 2). Thus, it has an important role in cell growth and survival [22]. The functional activity of AKT depends on RTKs (Receptor tyrosine kinases) which are overexpressed in multiple types of cancers. In addition, the up-regulation of some RTKs including EGFR, MET, and AXL are observed and introduced as oncogenic components in mesothelioma given these receptors are directly involved in the initiation and activation of pro-survival PI3K/AKT/mTOR signaling pathway, leading to MMe cell viability and survival [23–25]. AKT phosphorylates and activates MDM2 protein at Ser166 and Ser186, conducting p53 degradation and cell growth [26]. Encouraging results demonstrated that the suppression of this signaling cascade increases the expression level of p53, p21, and MDM2 as well as MDM2 functional inactivation that is conducive to increased cell apoptosis and G1/G2 cell-cycle arrest and consequently reduces cell viability. Therefore, PI3K/AKT/mTOR suppression has attracted a lot of attention and there is some sort of evidence that it has positive effects on MMe therapy. Accordingly, the suppression of PI3K/mTOR by BEZ235 is suggested to have a greater anti-proliferative effect on all mesothelioma cell lines compared with other inhibitors like mTOR (RAD001) and (MEK) U0126 inhibitors. As a result, targeting PI3K/AKT/mTOR reduces 70–80% of the cell viability in mesothelioma cell lines [27]. However, in vivo studies are needed to confirm these in vitro results.

As we mentioned above, targeting the PI3K/AKT/mTOR signaling pathway as a therapeutic strategy improves MPeM tumor shrinkage. On this basis, a clinical trial study investigated the role of apitolisib as a PI3K-mTOR inhibitor in two young female MPeM patients. Both of them are still alive. Saoirse O Dolly et al. implemented combinational therapy for both patients. Platinum-pemetrexed chemotherapy was conductive to disease stabilization and apitolisib led to tumor shrinkage. Also, results showed that, in one of the patients, apitolisib alleviated both symptoms and CA-125 (tumor marker) and in another one, this drug led to a reduction of tumor volume. This data demonstrates that apitolisib has a considerable anti-tumor effect in both patients through the inhibition of PI3K-mTOR [28].

Besides, p-mTOR expression is confirmed to influence MPM prognosis. P-mTOR is positively related to the early stage of mesothelioma and its positive rate experiences a significant decline in MPM late stage [29]. Taken together, the suppression of mTOR could improve both prognosis and cisplatin/pemetrexed effectiveness in MPM patients [30]. CDKN2A/ARF as a tumor suppressor gene encodes p16INK4a and p14ARF. These two cyclin-dependent kinase inhibitor proteins involve in cyclin D1/CDK4/6 inhibition and p53 activation, respectively, that overall lead to cell cycle arrest and apoptosis. CDKN2A/ARF gene is frequently (70%) inactivated in MPM patients. In this regard, Palbociclib is tested and qualified as a selective drug to mediate cell cycle arrest through the direct inhibition of CDK4/6 and p21 induction [31]. But this drug increases AKT phosphorylation and PI3K/AKT/mTOR activation. This prosurvival signaling pathway negatively regulates p53 protein by MDM2 accumulation, leading to cancer development. Therefore, it has been revealed that the combination of palbociclib with two PI3K/AKT/mTOR inhibitors, BEZ235 and BYL719, results in p53 and p21 accumulation, leading to the inhibition of cell proliferation and induction of cellular senescence in MMe cells [32]. This regimen can be applied to rat models to validate this in vitro demonstration. However, autophagy as an intracellular degradation system provides new building blocks and energy for cellular homeostasis and cell survival. To escape cell death, some cancer cells take advantage of autophagy. About this issue, PI3K/mTOR inhibitors (NVP-BEZ235 and GDC-0980) in MPM cell lines are a cause of autophagy induction and subsequently cell cycle arrest and cell death resistance. As a result, the combination of PI3K/mTOR inhibitors and CQ (Chloroquine) (autophagy blocker) should be considered to prevent cell death resistance in MPM [33].

Taken collectively, PI3K/AKT/mTOR inhibitors can increase the potency of MMe cell death and can be effective agents in improving MMe therapy in either single or combinational therapies.

### MAPK signaling pathway

The mitogen-activated protein kinases (MAPKs) are serine-threonine protein kinases that are involved in different kinds of cellular processes like proliferation, differentiation, apoptosis or survival, and inflammation [34]. MAPK pathway is generated by stimuli, after that MAP3Ks activate MAP2Ks and consequently provoke the activation of MAPKs which phosphorylates transcription factors c-Jun, c-Myc, and ATF2 responsible for the induction of mentioned cellular processes [35]. The involvement of the RAF/MAPK pathway in MMe progression has been studied widely [36]. However, further studies can be helpful to understand the molecular mechanism and genes expression related to Ras/MAPK signaling in MMe progression.

The same as pI3K/AKT/mTOR pathway, RAF/MAPK pathway activation depends on RTKs [37] which upregulate in MMe cells. RTKs inhibition results in proliferation inhibition in mesothelioma through the suppression of the PI3K/AKT and the RAF/MAPK pathways. The contribution of these two pathways in cancers progression has been proved in a huge number of studies, which attract attention to targeted combinational therapy. Moreover, there is a general agreement that both the PI3K/AKT and the RAF/MAPK signaling pathways are associated with MMe initiation and progression [38–41]. Thus, the application of PI3K and MAPK inhibitors together plays a synergistically more anti-proliferative role in MPM by the induction of cell apoptosis and cell cycle arrest at the G1/G2 phase [27]. According to the above findings, there may be coordination between these two oncogenic cascades and they can be inhibited together to alleviate MMe development.

It was observed that Pirfenidone alone or with cisplatin downregulates both MAPK/ERK and AKT/mTOR pathways which induce MPM cell proliferation. Both in vivo and in vitro results of this study show that Pirfenidone plays a significant role in improving the effectiveness of cisplatin through the modification of extracellular matrix-like changing the expression level of collagen genes, leading to accelerating the drug delivery to tumor tissue [42]. In addition, a higher level of p21-activated kinases (PAKs) as serine/threonine kinases participates in cancer development due to the regulation of cellular processes like proliferation, migration, gene transcription, and cell survival [43]. PAK1 enhances MAPK signaling functional activity and consequently cell survival, proliferation, and tumorigenesis by the phosphorylation of Raf-1 and MEK1. In this regard, the MAPK signaling pathway is the downstream effector of PAK in MMe. Furthermore, the tumor suppressor gene NF2 is frequently mutated in MPM. There is a negative correlation between NF2 and PAK signaling. Therefore, in NF2-mutated-mesothelioma cells, PAK signaling upregulation has been expected. So, pharmacological PAK suppressors can be an efficient strategy to prevent MMe tumor development through the alleviation of the Raf-MAPK signaling pathway [44].

In addition, Ras genes mutations are common in different types of human cancers [45]. Although Ras mutation is not observed in MMe cell lines and tumors, the Ras signaling pathway is activated through other mechanisms, leading to the improvement of MMe cancerous behavior. Therefore, the activation of Ras signaling occurs in a variety of human malignancies like MMe, resulting in the stimulation of cell proliferation. MAPK, PI3K/AKT, and JNK are introduced as the downstream kinases of the Ras cascade. Also, previously we mentioned that the suppression of these downstream kinases of the Ras pathway has an anti-proliferative effect on mesothelioma cell lines. In conclusion, it has been suggested that Ras/MAPK functional activity provokes mesothelioma cell differentiation from non-transformed cells [46]. It is inferred from these studies that the MAPK signaling pathway would be able to open new windows in MMe therapy.

### Calcium signaling

Calcium ions (Ca2 +) are practically involved in the progression of human cancers. New evidence shows that one of the characteristics of MMe cells is the alteration in the expression and activation of calcium signaling [6]. The intracellular concentration of Ca2 + is lower than the extracellular space and Ca2 + is stored in the cell. When calcium is released in the cell, it plays a role as a second messenger and initiates a signaling pathway, leading to tumor development and the induction of malignant behaviors [47]. However, the exact mechanism of action and signaling pathways related to Ca2 + have not been investigated yet in MMe and further examinations can be helpful to shed more light on the involvement of Ca2 + in MMe development.

Meanwhile, ion channels are responsible for Ca2 + homeostasis. On this basis, T-type Ca2 + channels as low voltage channels take part in Ca2 + transportation. The overexpression of T-type Ca2 + channels has been reported in MMe cell lines. Also, it has been proved that T-type Ca2 + channel blockers have general anti-cancer properties to alleviate cell growth and provoke cell death. It has been suggested that blocking these channels could be considered a therapeutic subject for MMe treatment [6, 48].

**Fig. 3:**
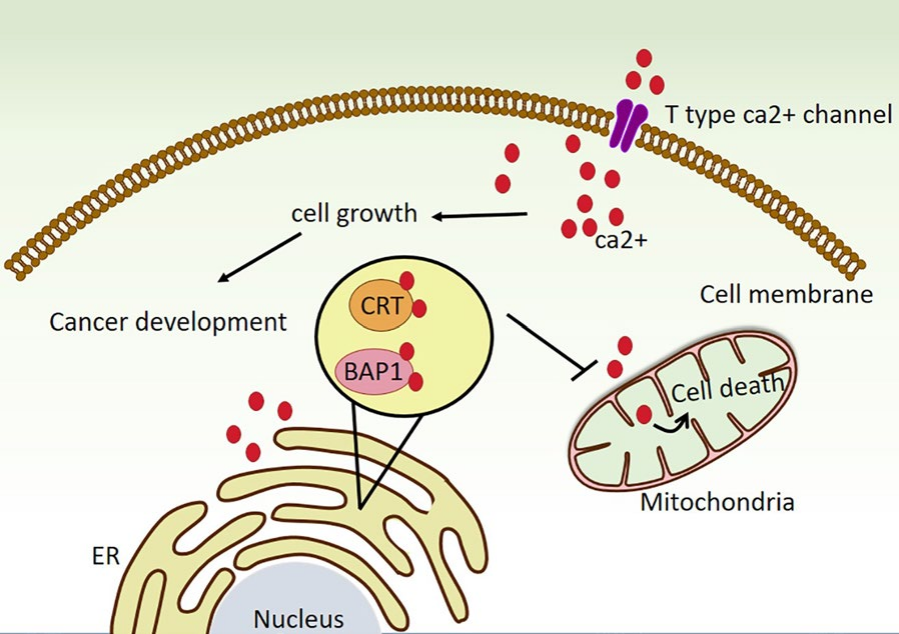
Alteration in expression and activation of calcium signaling in mesothelioma cells. T-type Ca2 + channels overexpression increases Ca2 signaling and tumor development. CRT and BAP1 overexpression keep Ca^2+^ in the ER, reducing Ca2 + uptake into the mitochondria and inhibiting cell apoptosis. *BAP1* BRCA1 associated protein 1, *CRT* Calretinin, *E;* endoplasmic reticulum

Moreover, Calretinin (CRT) is a calcium-binding protein controlling intracellular calcium signaling. There is some initial evidence that CRT links to mesothelioma transformation and mesotheliomagenesis through the regulation of intracellular Ca^2+^ homeostasis or Ca^2+^ buffering [6]. CRT up-regulation detected in MMe is in contrast to normal mesothelial cells. Thus, it is introduced as one of the MMe markers [49]. The higher expression of CRT causes a Ca^2+^ handling impairment, leading to the reduction of Ca^2+^ uptake into the mitochondria and consequently the inhibition of mesothelial cells apoptosis. However, more investigations are needed to clarify this evidence [50]. As mentioned above, BRCA1 associated protein 1 (BAP1) gene is commonly mutated in MMe. BAP1 is a deubiquitinase enzyme is located in the endoplasmic reticulum (ER) and indirectly controls Ca2 + flux release from the ER to the mitochondria. Ca2 + released from the ER plays a complementary role in apoptosis regulation by the mitochondria. On the other side, exposure to asbestosis provokes ER Ca2 + release which influences the mitochondria and ER cross talk and intracellular Ca2 + concentration to determine cell fate [6, 51] (Fig. 3). As a result, targeting calcium signaling could be a novel approach in the diagnosis and treatment of MMe patients. Based on the above findings, T-type Ca2 + channel, CRT and BAP1 can be selecting candidates to be targeted for finding an effective approach in MMe therapy.

## DNA repair machinery in mesothelioma

**Fig. 4:**
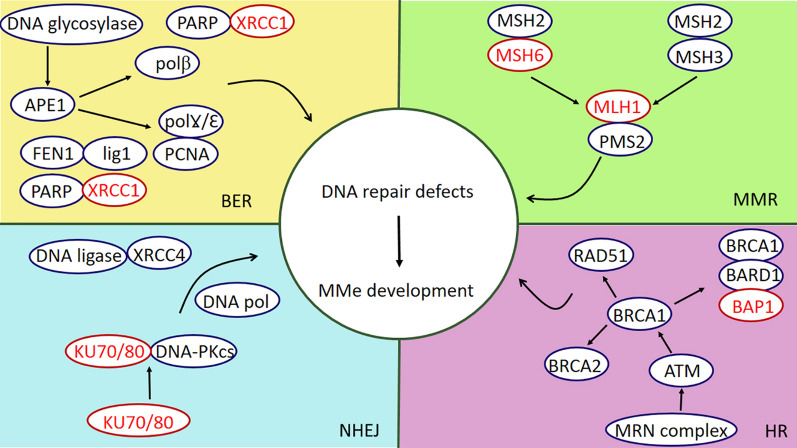
The association between mutations in DDR components and MMe development after DNA damage. XRCC1, MSH6, MLH1, KU70/80, and BAP1 are frequently mutated among BER, MMR, NHEJ, and HR repair pathways, leading to MMe development. *MMe* Malignant mesothelioma, *BER* base excision repair, *MMR* miss match repair, *NHEJ* nonhomologous end-joining repair, *HR* homologs recombination

The MMe risk factors have effects on genomic alterations mainly with DNA damage causing this cancer; for instance, reactive oxygen species (ROS) and reactive nitrogen species (RNS). Asbestos fibers, as the main cause of MMe, produce both ROS and RNS directly and indirectly [52]. Although genomic damage can be the primary cause of MMe, another essential second arm assisting the creation and development of MMe is the DNA repair system. The DNA repair machinery system eliminates different types of DNA damage through a complex group of 5 distinct pathways: Base excision repair (BER), Nucleotide excision repair (NER), DNA mismatch repair (MMR), Homologous recombination (HR), and Non-homologous end joining (NHEJ) [53]. Therefore, defects in DNA repair genes reduce DDR functional activity and obtain cancerous behavior like escaping from apoptosis and subsequently increase malignancy’s predisposition like MMe. BAP1, XRCC1, PALB2, BRCA1, FANCI, ATM, SLX4, BRCA2, FANCC, FANCF, PMS1, and XPC are the most common mutated DNA repair genes in MMe [54, 55] (Fig. 4). Indeed, DNA repair has dual effects on cancer prevention and development. In a normal situation, the DNA repair machinery appears to repair the damages and stop the formation of cancerous cells. On the other side, when cells acquired cancerous features, they use a DNA repair mechanism to stop apoptosis and survive. In this regard, it is important to evaluate the mutations and gene expression alterations in the DNA repair process to figure out the underlying mechanisms of cancer development. A study in 2018 revealed that 83% of germline mutations in MMe cases were in charge of DDR, and exclusively 50% in the HR pathway. This evidence shows the importance of DNA damage response defects in MMe [56]. As a result, single-nucleotide polymorphisms (SNPs) have been widely investigated in MMe patients [57]. BER, one of the DNA repair systems, corrects small base errors without alteration in the DNA helix. This system contains two patches, a short patch and a long patch. In the beginning, DNA glycosylase and apurinic/apyrimidinic endonuclease 1 (APE1) recognize, remove and create a strand incision, respectively. Then, it can follow one of the patches. In the short patch, scaffold protein X-ray cross-complementing group 1 (XRCC1) connects DNA polβ and DNA ligase3 to replace a single correct base. However, in the long patch the complex of DNA polƔ/Ɛ, proliferating cell nuclear antigen (PCNA), the flap endonuclease 1 (FEN1), and DNA ligase1 syntheses 2–10 bases and correct the damage [58]. XRCC1 is located in chromosome 19q13.2 with seventeen exons. Polymorphisms of the XRCC1 genome cause to change of amino acids ordered and directly affect its scaffold function and interaction with other DNA repair components, enhancing the risks of malignancies [59, 60]. In this regard, the SNP XRCC1-399Q just in association with asbestos exposure is correlated with MPM. In addition, excision repair cross-complementing group 1 (ERCC1) protein, together with xeroderma pigmentosum group F complementing protein (XPF), are involved in mammalian cells’ DNA repairs systems such as NER, HR, NHEJ, and BER. Taken together, a case–control study revealed the association between XRCC1 R399Q and ERCC1 N118N in MPM patients [61, 62]. In addition, ERCC1 rs11615 polymorphism is correlated with asbestos exposure and MMe development, while ERCC1 rs3212986 polymorphism has a protective role in MMe progression [63].

PARP1 is a DNA damage sensor that plays a considerable role in repair pathways, including BER, NHEJ, and HR, and also has functions in apoptosis and cell death induction [64]. PARP expression and activation increase during asbestos exposure in human mesothelial cells. Therefore, PARP1 causes cell death induction during asbestos exposure [65]. On the other side, the expression level of PARP1 in MPM patients has proved to be higher than in the control group, while it appears to have lower activity leading to DNA repair inefficacy and malignant transformation [66]. Regarding these results, future studies are required to elucidate the exact PARP1 situation in MMe progression.

The mismatch repair (MMR) system is one of the major DNA repair pathways mediated by MSH2, MSH6, MSH3, MLH1, and PMS2. In this pathway, MSH2 and MSH6 heterodimer or MSH2 and MSH3 heterodimer recognize and bind to small insertion/deletion loops and larger insertion/deletion loops, respectively. Moreover, a heterodimer of MLH1 and PMS2 is required for completing this complex and repairing the damages [67, 68]. MLH1, MSH2, MSH6, and PMS2 mutations have been reported in MPeM. Additionally, although MMR impairment is not common among MPM patients (just 2.5% of cases); MLH1, MSH6, and MLH3 mutations have been seen in the biopsy of the patients. MLH1 mutations and inactivation are more frequent than MSH6 and MLH3 [69]. Another critical genome repair pathway is HR which demonstrates a central role in repairing double-strand break (DSB). Following a DSB, the MRN (MRE11, RAD50, NBS1) complex binds to the damage site, increases ATM activation, and activates a set of downstream proteins like BRCA1. Then, recombinase RAD51 is recruited by BRCA1 and mediates recombinase activity to complete the repair process [54]. In addition, the BRCA1/BARD1 complex regulates nucleosome and chromatin in the HR pathway [70]. Increased expression of proteins involved in HR, such as RAD50, RAD54L, RAD21, and BRCA2, or deletion of the gene region in MMe cells, indicates the importance of these proteins in MMe pathology [69]. BRCA1 associated protein 1 (BAP1) is a tumor suppressor protein involved in the regulation of fundamental cellular processes such as proliferation, differentiation, and apoptosis. Also, there is some encouraging evidence that BAP1 is a member of the BRCA1/BARD1 complex participating in the DDR pathway [54]. In addition, it has been reported that BAP1 contributes to other cellular mechanisms like cell cycle progression, genome stability, and DNA repair. Individuals who carry BAP1 mutations are considered at high risk in susceptibility to MMe development [71]. On this basis, as we mentioned above, BAP1 is one of the most frequent mutated genes in the MMe. For example, experimental analysis of Sanger sequencing in two studies shows 23% and 20% BAP1 mutations in US MMe patients [72, 73]. In another study, 60% of MMe samples showed BAP1 nuclear staining loss by using IHC (immunohistochemical) method [74]. Also, 14 out of 23 MMe Japanese patients’ specimens showed BAP1 mutation [75]. Moreover, Masaki Nasu et al. reported that more than 60% of MMe biopsies have somatic BAP1 gene alterations [76]. Taken together several studies have declared that, 30–60% of MMe cases show BAP1 genome mutations, including point mutations and exon deletions, conducting to BAP1 loss of function and increasing MMe risk [69].

Finally, NHEJ is the fifth DDR pathway in cells. KU70/80, DNA-PKcs, XRCC4-ligase4 complex, and certain DNA polymerases are introduced as the core components of NHEJ. KU70/80 is the first complex in the NHEJ pathway being responsible for break recognition [77]. A study shows a cancer-driver Ku70 point mutation conducting amino acid substitution in MPM specimens [78]. Overall, after asbestos exposure, the mutations, polymorphisms, and activation of DNA repair components like XRCC1, ERCC1, PARP, MLH1, MSH2, MSH6, PMS2, RAD50, RAD54L, RAD21, BRCA2, BAP1, and Ku70 are involved in the progression of MMe; because these changes enable cancer cells to repair the damages and escape cell death pathways, leading to MMe development. This valuable evidence can help find new strategies for MMe treatment. On this basis, in the following section, we will review the latest findings of DNA repair targeted therapy in MMe.

## Targeting DNA repair as a therapeutic approach in mesothelioma

The average treatment for MPM patients most generally is surgery accompanying chemotherapy and radiotherapy. But only a few are eligible for these treatments, some show strong resistance to chemotherapy, and few have the proper conditions for surgery [79]. Since DNA repair mutations have been confirmed in MMe development, studies have shown that targeting DNA repair can be a promising way to treat MPM. Although a mixture of DNA repair pathway inhibitors including PARP, DNA-PK, ATR, ATM/ATR, or the MRN complex inhibitors have been used alone or in combination with other antitumor drugs to treat tumors, among them, PARP inhibitors (PARPi) play significant roles in dealing with MPM [8, 69]. The PARP inhibitor has been studied in many other tumors [80]. Niraparib, olaparib, talazoparib, and rucaparib have already been approved for the therapy of breast, pancreatic, and ovarian cancer, which reacts against tumors containing mutations in the BRCA or other HR genes [8]. According to this view, the theory that the PARP inhibitor could have good potential for the treatment of MPM patients has been developed, at least for patients with HR gene changes, especially BAP1. This theory has been tested on various MPM cellular models. The PARP inhibitor had a toxic effect on several MPM cell lines, although this effect was independent of the status of the BAP1 mutation [72, 81]. Studies show that PARP inhibitors have synthetic lethal interaction with HR-deficient tumors. The binding of PARP1 to ssDNA disruptions generated in BER forms is the basis of synthetic lethal interaction with HRD. When BER cannot correct ssDNA failures, these single-stranded failures become double-stranded DNAs. Cells with the HR repair system can easily repair these failures, but in the event of a defect in this system, the cells rely on the NHEJ repair system. Finally, due to its error-prone nature, genomic instability occurs and prevents cancerous cells progression and provokes cell death. Studies show that PARP inhibitors trap the PARP enzyme at the site of DNA damage and inhibit essential cell functions such as transcription and DNA repair. In addition, the PARP-DNA complex trapped in cells with defective HR is lethal and helpful to prevent cancer development [8]. CCDC6 affects the response of MPM models to PARP inhibitors. CCDC6 is one of the components of ATM essential for HR’s function in repairing DNA damage. According to a study, the silencing of CCDC6 in MPM cells containing BAP 1 mutant, increases PARPi sensitivity. Mutant BAP1 in combination with CCDC6 silencing enhanced the HR-DNA repair defects and sensitivity to olaparib. As a result, CCDC6 could be considered a predictive marker for PARP inhibitor treatment in MPM patients [82]. PARP1 inhibitor requires some proteins, such as SLFN11 to function. Following DNA damage, SLFN11 is considered one of the DNA/RNA helicase blocking replication forks. Some research shows that when SLFN11 is inactivated, resistance to iPARP1 inhibitor in several tumors, including MPM is created [83]. In a study, PARP1 level in response to PARP1 inhibitor significantly reduced the survival of MPM cells in a rucaparibin dose-dependent manner. These findings can be explained as the formation of cytotoxic DNA-PARP-PARPi triple complexes, an important component of how PARP1 inhibitor works to trap molecule PARP1 in DNA. Decreasing the concentration of PARP1 reduces the complex formation and ultimately reduces activity. To prove this, an increase in PARP1 through exposure to hydrogen peroxide makes human mesothelial cells highly sensitive to rucaparib in vitro [84]. Studies show that PARP inhibitor increases the cytotoxic effects of ionizing radiation and chemotherapeutic agents such as alkylating agents [84]. According to studies, when DNA is damaged by asbestos, the rate of PARP increases for DNA repair [85]. Various studies have shown that the inhibition of PARP increases AKT phosphorylation and also reduces the ability of cancer cells to repair DNA damage and survive [86]. Interestingly, the activation of AKT with PARP1 inhibitor is not able to modulate pre-survival signals. It seems that the effective downstream pathway in the mTOR level has been interrupted. Pharmacological inhibition of PARP increases NAD + content and activates SIRT1, indicating that PARP1 acts as a barrier to SIRT1 activity by limiting NAD + [87]. According to studies, SIRT1 has a role in modulating AKT [87]. SIRT1 shows an inverse relationship between acetylation AKT and phosphorylation in MPM cells treated with PARP1 inhibitor. SIRT1 can also modulate the phosphorylation of mTOR [88]. Research has shown that the damage to DNA caused by platinum compounds causes PARP1 to bind to DNA for repair. The combined effect of PARP1 inhibitor and platinum drugs is expected to be effective in curing the disease; because when the damages are accumulated and the DNA repair is prevented, cancer cells are doomed to death [84]. There are several in vitro observations showing the synergistic effect of anticancer drugs in MPM cell lines, for example, olaparib plus cisplatin, which has been effective in increasing the death of MPM cells mutating in BAP1. Also, the combination of rucaparib and cisplatin in different concentrations has been effective in the death of three MPM cell lines [84, 89].

Trabectedin is another drug that is being tested in the laboratory for the treatment of MPM by targeting the DNA repair system [90]. It has been already approved for ovarian cancer and soft tissue sarcoma, and its derivative lurbinectedin (PM01183). According to studies, NER deficient cells show abnormal resistances, while HR deficiency causes hypersensitivity to trabectedin [91, 92]. For example, studies on cells with TC-NER deficiency show that these cells are 2 to 10 times less sensitive to trabectidine than other anticancer agents, such as platinum compounds [93, 94]. TC-NER detects damage to the DNA spine and for repairing these injuries uses a variety of factors. Although the additional compounds of trabectidine are not repaired by the NER, they interfere with the NER mechanism and prevent the repair of certain NER substrates. According to studies, this may be due to the direct interaction of trabectidine with the components of the NER [94].

Studies have shown that HR is directly related to the response to trabectidine treatment. HR-defective cells are 100 times more sensitive to this drug. HR deficiency was associated with the persistence of unrepaired DSBs along with the S phase of the cell cycle pathway with apoptosis [90, 95]. The sensitivity of cells with HR deficiency has been clinically confirmed. This drug is very active in patients with recurrent ovarian cancer who have a BRCA1 mutation [96]. This result has also been observed in patients treated for metastatic breast cancer with a BRCA1/2 mutation [97]. Trabectedin binds to the DNA small groove and interacts with proteins that bind to DNA, such as transcription factors and DNA repair proteins. In addition to affecting the environment around the tumor, it also has a direct effect on the survival and proliferation of cancer cells [98]. Some studies show that trabectidine not only reduces the number of tumor-associated macrophages (TAM) but also reduces the production of several inflammatory and angiogenic factors including interleukin 6 and vascular endothelial growth factor and chemokines such as CC motif chemokine ligand 2 [99]. Since the up-regulation of chemokine pathways generally lead to severe symptoms [100], these features, along with some evidence of activity in preclinical mesothelioma models encourage Trabectedin evaluation as a treatment option in MPM.

Immune checkpoint blockade ICI, which is an immune-regulating factor, that enhances the latent immune response kept in check by the tumor, is also under consideration against MPM. According to studies, the inactivation of the MMR genes such as mutL homolog 1 gene (MLH1), PMS1 homolog 2, mismatch repair system component gene (PMS2), mutS homolog 2 gene (MSH2), and mutS homolog 6 gene (MSH6) cause microsatellite instability of the MSI. The most common of these translated proteins form heterodimers fixing DNA lesion, are mutL homolog 1 (MLH1) / PMS1 homolog 2, mutation repair system M2 (PMS2) and mutS homolog 2 (MSH2) / mutS homolog 6 (MSH6). Thus, mutations in the genes of these proteins lead to immune mutations, and the MMR deficiency predicts the clinical advantage of ICI in many tumors; but because the MMR defects are rare in MPM patients and the MSI phenotype is absent, it is not yet related to the response of patients to ICI [101, 102]. Recent research suggests that MPM patients with BAP-1 deficiency may show a distinct molecular subset with increased sensitivity to ICI therapies. According to these studies, tumors with BAP1 haploinsufficiency form a distinct molecular subset determined by distinct templates of chromatin regeneration, gene expression, immune checkpoint receptor activation, DNA repair pathways, and an inflammatory tumor microenvironment. BAP-1 deficiency causes damage to DNA as well as defects in the DNA repair mechanism [103]. Defects in the DNA repair mechanism cause genetic instability and disruption of the tumor environment [104]. This defect leads to increased secretion of cytokines such as interferons, which in turn increases the delivery of tumor antigen to lymphocyte T to destroy the tumor. Tumors also escape from the immune system by increasing immune checkpoint receptors. In this defect, the expression level of immune checkpoint receptors increases suggesting the use of immune checkpoint inhibitors to treat this subset of MPM [105, 106]. Overall, when DNA repair processes are disrupted, cancer cells cannot repair the damages and prevent apoptosis. Many of these laboratory studies, which target DNA defects in the treatment of MPM cancer, have the potential to cure MPM in the future and may be involved in increasing the life expectancy and recovery of this disease along with primary therapies such as surgery, chemotherapy, and radiotherapy.

## Conclusion and perspectives

In this paper, we have discussed the involvement and dysregulation of survival signaling pathways including, Hedgehog signaling, PI3K/AKT/mTOR signaling, MAPK signaling, and calcium signaling in MMe progression. Also, encouraging evidence showed the important role of DNA damage and DNA repair in MMe development. The importance of DNA repair failure is to the extent that 83% of MMe cases mutations were in charge of DDR. Since DNA repair mutations are confirmed among the most significant alterations in MMe development, DDR regulation can be a promising approach to stop cancer cells progression and provoke apoptosis. Therefore, much attention has been drawn to targeting DDR as a treatment strategy for MMe treatment. However, we believe that there is a long way to achieve this goal and further investigations are required to prove the efficacy of this strategy.

## Data Availability

Data sharing is not applicable to this article as no new data were created or analyzed in this study.
